# Sleep‐Dependent Clearance of Brain Metabolites via the Glymphatic System: Implications for Alzheimer's Pathophysiology

**DOI:** 10.1002/brb3.71374

**Published:** 2026-04-14

**Authors:** Farshad Zare, Gulnora Shakhmurova, Jasur Rizaev, Aseel Smerat, Rosull Saadoon Abbood, Vinay Patil, Manoj Kumar‐Mishra

**Affiliations:** ^1^ Student Research Committee, School of Medicine Tabriz University of Medical Sciences Tabriz Iran; ^2^ Department of Biological Sciences National Pedagogical University of Uzbekistan named After Nizami Tashkent Uzbekistan; ^3^ Department of Public Health and Healthcare Management, Rector Samarkand State Medical University Samarkand Uzbekistan; ^4^ Hourani Center for Applied Scientific Research Al‐Ahliyya Amman University Amman Jordan; ^5^ Medical Laboratory Techniques department, College of Health and Medical Technology University of Al‐Maarif Anbar Iraq; ^6^ Long Island University, Brooklyn New York USA; ^7^ Salale University Fitche Ethiopia

**Keywords:** Alzheimer's disease, amyloid‐beta, aquaporin‐4, brain clearance, cerebrospinal fluid, glymphatic system, sleep, tau

## Abstract

**Purpose:**

This review aims to examine how sleep‐dependent glymphatic function contributes to the clearance of brain metabolites involved in Alzheimer's disease (AD), with particular emphasis on amyloid‐beta (Aβ), tau, astrocytic aquaporin‐4 (AQP4), and emerging biomarkers of clearance‐related dysfunction.

**Method:**

A narrative review of recent mechanistic, preclinical, and human studies was conducted to synthesize current evidence linking sleep, glymphatic transport, and AD pathophysiology. Findings from animal experiments, diffusion MRI proxies such as diffusion tensor imaging analysis along the perivascular space (DTI‐ALPS), biomarker studies, and translational intervention research were integrated, with attention to sleep physiology, vascular dynamics, and neuromodulatory regulation.

**Findings:**

Available evidence indicates that sleep is an active physiological state that facilitates cerebrospinal fluid (CSF) exchange with interstitial fluid and promotes the removal of neurotoxic solutes from the brain. Glymphatic transport appears to be most active during non‐rapid eye movement sleep, particularly during slow‐wave activity, when interstitial space expands and CSF‐interstitial fluid exchange increases. Experimental studies show that sleep enhances the clearance of Aβ, tau, and related metabolites, whereas sleep disruption, aging, vascular dysfunction, and AQP4 abnormalities impair this process and may accelerate AD‐related pathology. Human evidence has also advanced, including DTI‐ALPS studies and a 2026 randomized crossover study reporting higher morning plasma amyloid and tau levels after normal sleep than after sleep deprivation, consistent with greater overnight brain‐to‐blood clearance during sleep.

**Conclusion:**

Sleep‐dependent glymphatic clearance is increasingly recognized as an important component of brain homeostasis and a plausible contributor to AD pathophysiology, with promising implications for biomarker development, prevention, and future therapeutic translation.

## Graphical abstract

Slow‐wave sleep promotes glymphatic transport through coordinated norepinephrine dynamics, vasomotion, and polarized AQP4‐dependent fluid exchange, thereby facilitating clearance of amyloid‐beta and tau. In contrast, sleep disruption, aging, vascular dysfunction, and AQP4 abnormalities impair this system and may accelerate Alzheimer‐related pathology.

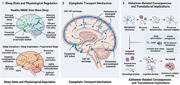

**Abbreviations**
AD: Alzheimer's disease
AQP4: Aquaporin‐4
Aβ: Amyloid‐beta
Aβ PET: Amyloid‐beta positron emission tomography
CBT‐I: Cognitive behavioral therapy for insomnia
CPAP: Continuous positive airway pressure
CSF: Cerebrospinal fluid
DTI‐ALPS: Diffusion tensor imaging analysis along the perivascular space
EEG: Electroencephalography
ISF: Interstitial fluid
LC: Locus coeruleus
MCI: Mild cognitive impairment
MRI: Magnetic resonance imaging
NCT: National Clinical Trial
NREM: Non‐rapid eye movement
PET: Positron emission tomography
pTau217: Phosphorylated tau 217
SWA: Slow‐wave activity
tACS: Transcranial alternating current stimulation
WMH: White matter hyperintensities

## 1 Introduction

Alzheimer's disease (AD) is the leading cause of dementia and is characterized by progressive cognitive decline accompanied by amyloid‐beta plaque deposition, tau pathology, synaptic dysfunction, and neurodegeneration (Alzheimer's Association 2024; Tenchov et al. 2024). Although these pathological hallmarks are well established, the mechanisms that drive disease onset and progression are still not fully understood. Traditional models have largely emphasized abnormal protein production and aggregation, yet the limited clinical benefit of many amyloid‐ and tau‐targeting therapies has increased interest in upstream processes that may shape the neurodegenerative environment more broadly (Karran and De Strooper 2022; van Dyck et al. 2023).

Accordingly, contemporary frameworks increasingly conceptualize AD as a multifactorial disorder in which amyloid and tau pathology interact with neuroinflammatory, vascular, and infection‐related mechanisms, with the relative contribution of each pathway potentially varying across disease stages and individuals (Grobler et al. 2023; Gunday and Deniz 2026). Within this broader view, impaired clearance of toxic metabolites has emerged as a particularly important contributor to disease development. In recent years, the glymphatic system has gained attention as a compelling model for understanding how the brain removes soluble waste (Iliff et al. 2012; Louveau et al. 2015). This glia‐dependent perivascular transport pathway enables cerebrospinal fluid (CSF) to enter along periarterial spaces, exchange with interstitial fluid (ISF) within the parenchyma, and exit through perivenous and meningeal drainage routes. Astrocytic aquaporin‐4 (AQP4) channels, concentrated at vascular endfeet, are thought to play a central role in supporting this exchange (Mestre et al. 2017). The activity of this system is influenced by several physiological factors, including vascular pulsatility, astroglial integrity, circadian timing, and especially sleep state. Early experimental studies showed that natural sleep and some anesthetic conditions markedly enhance CSF‐ISF exchange, providing a plausible mechanistic explanation for why sleep supports brain homeostasis beyond its established roles in synaptic recalibration, hormonal regulation, and memory processing. This is particularly relevant to AD because the relationship between sleep and neurodegeneration appears to be bidirectional (Zahran et al. 2026). Poor sleep quality, chronic sleep deprivation, insomnia, and sleep‐disordered breathing have been associated with greater amyloid burden, tau abnormalities, and increased risk of cognitive decline, while early AD‐related changes can in turn disrupt sleep architecture and circadian regulation (Spira et al. 2014).

Growing evidence from animal models, human imaging studies, and biomarker investigations suggests that glymphatic dysfunction may contribute to the accumulation of amyloid‐beta and tau, accelerate neuronal vulnerability, and interact with broader pathological processes in AD (Zhao et al. 2025). At the same time, advances in neuroimaging and physiological monitoring have begun to improve in vivo assessment of clearance‐related biology, while emerging therapeutic strategies aim to restore or enhance these pathways through sleep modulation, vascular support, neuromodulation, and astroglial targeting (Gazerani 2025). In this review, we examine the mechanistic and translational links between sleep‐dependent glymphatic clearance and AD pathophysiology. We first summarize the physiology of glymphatic transport and its regulation by sleep, vascular dynamics, and neuromodulatory signals. We then evaluate evidence that sleep influences amyloid and tau homeostasis across experimental and clinical studies. Finally, we discuss imaging biomarkers, emerging interventions, clinical implications, and unresolved controversies, with particular attention to the strengths and limitations of the current human evidence base.

## 2 Mechanistic Basis of Sleep‐Dependent Glymphatic Clearance

### 2.1 Physiology of the Glymphatic System

The glymphatic system is generally described as a brain‐wide perivascular network that supports fluid and solute movement between CSF and interstitial fluid. In this model, CSF enters along periarterial spaces, exchanges with interstitial fluid in brain tissue, and then exits along perivenous and meningeal drainage pathways. Several forces contribute to this process, including arterial pulsation, vasomotion, and pressure gradients across fluid compartments (Ding et al. 2023).

Astrocytes are central to this framework. Their perivascular endfeet form an interface between vascular and parenchymal compartments, and AQP4 is highly enriched in these membranes. Experimental work has shown that loss of AQP4 reduces CSF‐interstitial fluid exchange and slows clearance of injected solutes, including Aβ (Gomolka et al. 2023). Importantly, dysfunction may arise not only from AQP4 deletion but also from loss of perivascular polarization. Age‐related or disease‐related mislocalization of AQP4 may therefore impair clearance even when total protein expression is preserved (Simon et al. 2022). Although the glymphatic model has gained broad attention, the broader brain clearance field remains conceptually complex. Meningeal lymphatics, perivascular basement membrane drainage, diffusion, dispersion, and advection likely all contribute to solute movement. This matters because the relative contribution of each pathway may differ by molecule, brain region, age, vascular state, and disease stage. Thus, while the glymphatic framework remains highly useful, it should be interpreted as part of a larger clearance network rather than as the sole mechanism (He and Sun 2025).

### 2.2 Sleep, Neuromodulation, and Glymphatic Enhancement

A defining feature of glymphatic transport is that it is highly state‐dependent. During deep NREM sleep, extracellular space expands, noradrenergic tone falls, and CSF influx into the brain increases. In contrast, wakefulness is associated with higher norepinephrine levels, narrower interstitial space, and reduced convective exchange. This state dependence provides a plausible explanation for why sleep supports metabolic homeostasis (Jessen et al. 2015).

**FIGURE 1:**
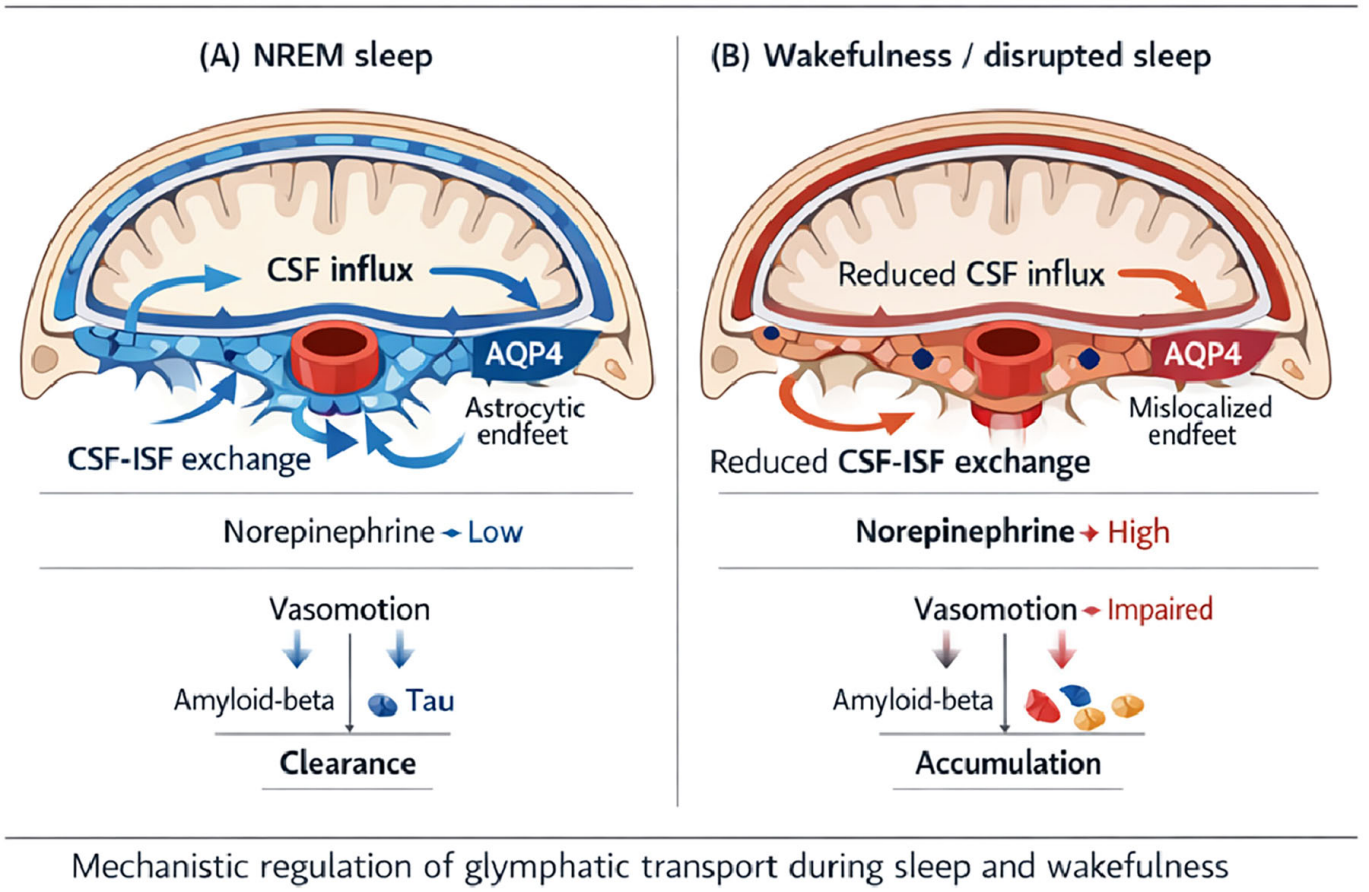
Sleep‐state‐dependent regulation of glymphatic transport. (A) NREM sleep supports glymphatic function through low noradrenergic tone, intact vasomotion, and polarized AQP4 expression at astrocytic endfeet, thereby enhancing periarterial CSF influx, CSF‐ISF exchange, and the clearance of amyloid‐beta and tau. (B) By contrast, wakefulness or fragmented sleep disrupts these processes through increased norepinephrine tone, impaired vascular dynamics, reduced fluid exchange, and AQP4 disorganization, resulting in inefficient waste efflux and retention of neurotoxic proteins relevant to Alzheimer's disease pathophysiology.

A foundational mouse study showed that natural sleep or anesthesia increased interstitial space volume by about 60%, accompanied by a marked increase in CSF‐interstitial fluid exchange. Subsequent work reinforced the idea that slow‐wave sleep is especially important for clearance. More recently, mechanistic studies have refined this picture by identifying a sleep‐linked neurovascular pump in which infraslow norepinephrine oscillations coordinate vasomotion and pulsatile CSF inflow (Xie et al. 2013). In mice, manipulation of locus coeruleus activity altered vasomotion and CSF dynamics, while zolpidem suppressed norepinephrine oscillations and reduced glymphatic flow. These findings suggest that not all sleep‐like states are biologically equivalent. A sedative may facilitate behavioral sleep while still dampening physiological drivers of clearance (Hauglund et al. 2025). This is an important translational point. Much of the earlier literature implicitly treated sleep and anesthesia as functionally similar in terms of glymphatic enhancement. The newer evidence suggests that the specific architecture and neurovascular quality of the state matter. Slow‐wave activity, low but dynamic noradrenergic tone, and intact vascular oscillations may be more important than unconsciousness alone (Benveniste et al. 2019) (Figure 1).

### 2.3 Glymphatic Clearance of Alzheimer‐Related Metabolites

One of the main reasons the glymphatic system has become so relevant to AD research is its apparent role in the removal of amyloid‐beta and extracellular tau from the brain. Experimental tracer studies in rodents have shown that soluble Aβ can move along perivascular clearance routes and that this process becomes less efficient when aquaporin‐4 function is disrupted (Dagum et al. 2026). Similar observations have been reported for tau, supporting the idea that glymphatic failure may contribute not only to protein retention but also to the amplification and spread of pathological aggregates (Lopes et al. 2024).

Additional work has strengthened the link between impaired glymphatic transport and the accumulation of Alzheimer‐related proteins. In aged mice, glymphatic Aβ clearance declines substantially, with earlier studies reporting a marked reduction that coincided with vascular stiffening and diminished perivascular AQP4 support. These findings highlight the dependence of efficient solute efflux on preserved astroglial organization and vascular compliance (Zhao et al. 2025). There is also growing interest in the possibility that glymphatic dysfunction interacts with APOE4, the strongest common genetic risk factor for sporadic Alzheimer's disease. APOE4‐associated astrocytic and neurovascular alterations may disturb perivascular homeostasis, weaken AQP4 polarization at astrocytic endfeet, and impair CSF‐interstitial fluid exchange (Ding et al. 2025). In human cohorts, APOE4 status may intensify the association between reduced glymphatic proxy measures and greater cerebral amyloid burden, although this relationship still requires further validation (Rajič Bumber et al. 2025).

Sleep shapes this biology at more than one level. On one hand, sleep appears to facilitate waste removal. On the other, sleep deprivation may promote the production or release of pathogenic proteins. Human CSF studies and stable isotope labeling experiments indicate that acute sleep loss can increase overnight Aβ concentrations, at least partly through increased production during wakefulness (Gosch Berton et al. 2026). Tau appears to be similarly sensitive to sleep disruption. Both experimental and human studies suggest that wakefulness and sleep loss elevate interstitial and CSF tau, while chronic sleep restriction can enhance tau seeding and propagation in animal models. Some evidence further suggests that tau clearance may be even more vulnerable than Aβ clearance when glymphatic transport is compromised, possibly because of tau's more complex biochemical behavior and solubility characteristics (Holth et al. 2019).

Human findings add an important translational dimension. A 2026 randomized crossover study comparing normal sleep with overnight sleep deprivation found that several morning plasma amyloid and tau species were higher after sleep than after deprivation. This pattern was interpreted as reflecting more effective overnight brain‐to‐blood clearance during sleep (Dagum et al. 2026). At first glance, this may seem counterintuitive, since sleep is often thought to reduce biomarker release. However, the result is biologically plausible if sleep simultaneously improves clearance efficiency. In that case, morning plasma concentrations would reflect the balance between production, release, and removal rather than any one process alone. Other human data also support the broader clearance hypothesis (Eide et al. 2023). Acute sleep loss has been associated with greater cortical amyloid signal in cognitively normal adults, while multimodal imaging studies suggest that lower ALPS values together with greater perivascular space burden are linked to higher amyloid and tau tracer uptake. Related observations from idiopathic normal pressure hydrocephalus, a condition characterized by disturbed CSF dynamics, further support the idea that impaired fluid transport pathways can promote solute retention and Alzheimer‐like pathological changes (Stankeviciute et al. 2024).

Taken together, these findings support the view that glymphatic pathways are central to the handling of both Aβ and tau and that disruption of these routes may contribute directly to proteotoxic stress, neuronal vulnerability, and disease progression. This may be especially relevant during preclinical and prodromal stages, when clearance failure could precede or accelerate overt neurodegeneration. Representative human and translational studies linking sleep, clearance‐related measures, and Alzheimer‐related biomarkers are summarized in Table 1.

**TABLE 1:** Selected studies linking sleep, glymphatic function, and Alzheimer‐related biomarkers.

Study or model	Condition or context	Putative clearance‐related measure	Key finding	Ref.
Mouse	Natural sleep or anesthesia versus wakefulness	Two‐photon imaging; interstitial space assessment	Sleep expanded interstitial space and enhanced CSF‐interstitial fluid exchange.	(Xie et al. 2013)
Aged mouse	Aging‐related glymphatic decline	Aβ clearance through glymphatic pathways	Aging reduced Aβ clearance and was linked to vascular stiffening and reduced AQP4 support.	(Kress et al. 2014)
Human	Sleep deprivation versus sleep opportunity	Serial CSF sampling; stable isotope labeling	Acute sleep deprivation increased overnight Aβ concentrations.	(Lucey et al. 2018)
Human	Normal sleep versus overnight sleep deprivation, randomized crossover	Morning plasma amyloid and tau species; physiological modeling	Higher morning plasma amyloid and tau after sleep, consistent with greater overnight brain‐to‐blood clearance.	(Dagum et al. 2026)
Human	Acute sleep loss in cognitively normal adults	Amyloid PET imaging	Sleep deprivation was associated with greater cortical amyloid signal.	(Shokri‐Kojori et al. 2018)
Human insomnia cohort	Insomnia versus matched controls	DTI‐ALPS	Lower ALPS was associated with insomnia and altered network efficiency.	(Xiong et al. 2025)
Human multimodal imaging cohort	Reduced glymphatic proxy function in an AD‐related context	ALPS index; perivascular space burden; amyloid and tau imaging	Lower ALPS, together with greater PVS burden, was associated with higher amyloid and tau tracer uptake.	(Zhang et al. 2024; Hsu et al. 2023)
Human clinical bridge	Idiopathic normal pressure hydrocephalus with altered CSF dynamics	Tracer movement; CSF clearance‐related imaging	Disturbed fluid transport was linked to impaired clearance and AD‐like pathological overlap.	(Eide et al. 2020)

## 3 Evidence From Animal Models

Animal studies provide the strongest causal evidence linking glymphatic dysfunction, sleep disruption, and AD‐related pathology. In amyloid models, acute and chronic sleep deprivation increase interstitial Aβ levels and accelerate plaque deposition. In tau‐focused models, sleep loss increases interstitial and CSF tau and can worsen tau seeding and spread. Manipulation of AQP4 has further strengthened causal inference (Dagum et al. 2026). AQP4 knockout models show markedly reduced CSF‐interstitial fluid exchange and increased Aβ deposition. Beyond complete knockout, chronic pharmacologic inhibition of AQP4 has been shown to exacerbate tau propagation and worsen recognition memory in vivo, suggesting that impaired clearance is not simply correlated with pathology but may actively facilitate it (Kato et al. 2024).

Recent work has also expanded the range of relevant mechanisms. A 2025 mechanistic study identified sleep‐associated norepinephrine‐driven vasomotion as a driver of glymphatic inflow, while showing that zolpidem can suppress this process in mice (Hauglund et al. 2025). This adds neuromodulatory precision to the older literature and highlights the importance of vascular and neurochemical dynamics. Other experimental work suggests that gamma‐frequency entrainment, including 40 Hz stimulation, may engage clearance‐related pathways through AQP4‐dependent mechanisms and meningeal lymphatic changes (Hainke et al. 2025). Finally, vascular and systemic factors matter. Aging, hypertension, vascular stiffening, and obesity can reduce arterial compliance and vasomotion, impairing fluid movement and potentially worsening Aβ clearance. Conversely, chronic exercise may improve vascular health and restore AQP4 polarization, thereby indirectly supporting clearance biology (Sun 2015). Collectively, these studies suggest that glymphatic impairment in AD is unlikely to arise from a single defect. Rather, it may emerge from the convergence of sleep disruption, neuromodulatory imbalance, vascular dysfunction, and astroglial disorganization. Key experimental manipulations affecting glymphatic‐related mechanisms and Alzheimer's pathology are summarized in Table 2 (Table 2).

**TABLE 2:** Experimental manipulations affecting glymphatic‐related mechanisms and Alzheimer's pathology.

Intervention or model	Target or mechanism	Outcome on clearance biology or AD pathology	Ref.
Paravascular tracer mapping	Perivascular CSF flow and AQP4‐dependent exchange	Demonstrated paravascular solute clearance, including amyloid‐beta, reduced with AQP4 loss.	(Iliff et al. 2012)
**Sleep deprivation in amyloid models**	Increased wakefulness, altered neuromodulation	Increased interstitial amyloid‐beta and accelerated plaque deposition.	(Kang et al. 2009; Roh et al. 2014)
**Sleep deprivation in tau models**	Sleep loss and prolonged wakefulness	Increased interstitial and CSF tau and promoted tau seeding and spread.	(Holth et al. 2019; Zhu et al. 2018)
**Chronic pharmacologic AQP4 inhibition**	Glymphatic blockade in the tau propagation model	Increased tau aggregation and spread with worsened recognition memory.	(Lopes et al. 2024)
**Sleep‐associated norepinephrine vasomotion**	Infraslow norepinephrine oscillations and vasomotion	Identified a pump‐like mechanism driving CSF inflow; zolpidem reduced oscillations and glymphatic flow in mice.	(Hauglund et al. 2025)
**Gamma entrainment**	Neural oscillations interacting with AQP4 and lymphatic dynamics	Suggested clearance‐related mechanisms contributing to reduced pathology in models.	(Murdock et al. 2024)

## 4 Human Evidence and Biomarkers

### 4.1 Sleep, Alzheimer Biomarkers, and Human Kinetics

Human evidence linking sleep to AD biology has grown substantially, although it remains less direct than the animal literature. Polysomnographic and observational studies have shown that reduced sleep efficiency, diminished slow‐wave sleep, insomnia, and sleep apnea are associated with higher amyloid burden and worse cognitive outcomes (Kent et al. 2021). Acute sleep deprivation studies have reported increases in CSF Aβ and tau, and reduced NREM sleep has been linked to unfavorable Alzheimer biomarker profiles. The newer human literature has become more mechanistically ambitious (Liu et al. 2023). Stable isotope labeling studies support the idea that sleep deprivation can increase overnight Aβ concentrations through increased production. At the same time, the 2026 crossover study suggests that normal sleep may enhance brain‐to‐blood transport of amyloid and tau species. These findings are not contradictory. Rather, they imply that sleep influences both production and clearance, and that interpretation of fluid biomarkers requires attention to timing, compartment, and physiological state (Gosch Berton et al. 2026).

### 4.2 DTI‐ALPS and Imaging‐Based Proxies

Because direct measurement of glymphatic transport in humans remains challenging, DTI‐ALPS has become one of the most widely used noninvasive proxy measures. A number of studies have reported lower ALPS indices in patients with AD and mild cognitive impairment, and longitudinal findings suggest that reduced ALPS may be associated with faster amyloid PET accumulation, greater neurodegeneration, and a higher likelihood of clinical progression (Kamagata et al. 2022; Huang et al. 2024). In cognitively unimpaired older adults, lower ALPS values have also been linked to smaller volumes in Alzheimer‐vulnerable brain regions, higher plasma phosphorylated tau concentrations, and more rapid progression of white matter hyperintensities. Similarly, in primary insomnia, reduced ALPS has been associated with altered white matter network efficiency and poorer attention‐related performance (Chen et al. 2025).

At the same time, the interpretation of ALPS requires caution. Recent population‐based work indicates that ALPS is strongly influenced by age, vascular burden, and white matter abnormalities, raising the possibility that it reflects a broader mixture of vascular dysfunction, tissue microstructure, and fluid‐related changes rather than a specific readout of glymphatic flux alone (Satpathi et al. 2025). This has become one of the main unresolved issues in the field. While Alzheimer‐focused cohorts often show relationships between lower ALPS and amyloid burden, neurodegeneration, or disease progression, large community‐based studies suggest that these associations may weaken substantially after adjustment for vascular and white matter factors (Farina et al. 2025). For this reason, ALPS may be more informative when interpreted alongside complementary markers such as perivascular space burden, multimodal imaging measures, and fluid biomarkers rather than as a standalone index of glymphatic function. There is also emerging interest in whether APOE4 status modifies these relationships, since APOE4‐associated astroglial and neurovascular vulnerability may amplify the association between reduced glymphatic proxy measures and cerebral amyloid accumulation (Ding et al. 2025; Kang et al. 2025).

### 4.3 Tracer MRI and Emerging Physiological Measures

Intrathecal contrast‐enhanced MRI provides a more direct, although invasive, approach for evaluating tracer movement and clearance in humans. Studies using this method in selected neurological conditions and small Alzheimer‐related cohorts have shown altered tracer dynamics in cognitive impairment, supporting the idea that disturbed fluid transport may accompany or contribute to neurodegenerative pathology (Riseth et al. 2026). However, the invasiveness of these protocols limits their broader use in routine clinical assessment and makes them more suitable for specialized research settings than for large‐scale screening or longitudinal follow‐up. Additional insight comes from related clinical conditions marked by abnormal CSF dynamics (Reeves et al. 2020). Idiopathic normal pressure hydrocephalus has attracted particular interest in this regard, as studies in this population have shown impaired tracer movement and overlapping features of glymphatic dysfunction together with Alzheimer‐like pathological changes (Eide et al. 2020). These observations strengthen the broader view that failure of fluid transport pathways may be relevant across more than one neurological disorder and may help bridge mechanistic findings between altered CSF circulation and protein accumulation (Erhardt et al. 2025).

Emerging physiological tools may help narrow the gap between invasive imaging and scalable human assessment. A 2025 validation study reported that noninvasive electrical impedance spectroscopy could detect sleep‐associated changes in parenchymal resistance that paralleled extracellular volume‐related dynamics and predicted glymphatic solute exchange measured by contrast‐enhanced MRI (Dagum et al. 2025). Although this approach remains preliminary, it points toward a potentially useful strategy for future phenotyping of clearance biology in larger human cohorts. If validated further, such methods could complement MRI‐ and biomarker‐based approaches and improve the feasibility of tracking sleep‐dependent clearance in longitudinal and interventional studies (Costea et al. 2025).

## 5 Modulators, Interventions, and Therapeutic Translation

### 5.1 Sleep Physiology and Behavioral Interventions

Given the strong relationship between sleep and clearance, improving sleep quality is an obvious translational target. Interventions such as cognitive behavioral therapy for insomnia, treatment of sleep‐disordered breathing, and maintenance of stable sleep‐wake schedules may support clearance biology, although direct proof remains limited (Rossman 2019). Closed‐loop acoustic stimulation has attracted interest because it can enhance slow‐wave activity, the sleep phase most closely associated with clearance‐related physiology. A 2025 at‐home study in AD patients reported group‐level enhancement of slow‐wave activity and slow‐wave sleep, although the response varied substantially across individuals. Continuous positive airway pressure (CPAP) is another relevant intervention (Frias et al. 2025). A 2025 pilot study in patients with mild cognitive impairment, Alzheimer's biomarkers, and obstructive sleep apnea reported slower cognitive decline with 12 months of CPAP adherence, but the sample was small and causal inference remains limited (Frias et al. 2025).

### 5.2 Pharmacologic Modulation

Pharmacologic sleep manipulation is more complicated than it first appears. Mechanistic mouse work indicates that zolpidem can suppress norepinephrine oscillations and reduce glymphatic flow, suggesting that some hypnotics may impair the physiological drivers of clearance despite promoting sleep onset. Epidemiological evidence linking hypnotic use to dementia risk remains heterogeneous and susceptible to confounding, but the question deserves caution rather than dismissal (Hauglund et al. 2025).

Dual orexin receptor antagonists are particularly interesting because orexin signaling promotes wakefulness and has also been linked to amyloid dynamics. In a 2023 randomized controlled trial in cognitively unimpaired adults, acute suvorexant administration reduced CSF Aβ and phosphorylation at a specific tau site (Lucey et al. 2023). These data are promising, but longer trials are needed to determine whether such acute biomarker shifts translate into durable clinical or clearance‐related benefit. AQP4 remains another attractive target, although no clinically approved AQP4‐enhancing drugs currently exist. More realistic near‐term approaches may involve preserving AQP4 polarization indirectly through control of neuroinflammation, vascular dysfunction, and metabolic stress (García Ríos and Leon‐Rojas 2025).

### 5.3 Gamma Entrainment and Oscillatory Interventions

Gamma‐frequency entrainment, particularly at 40 Hz, has become one of the most intriguing intervention categories in this space. In mouse models, 40 Hz stimulation has been associated with reduced amyloid pathology, and mechanistic work suggests that AQP4‐dependent effects and meningeal lymphatic changes may contribute. Human studies are still early but increasingly diverse (Chan et al. 2022).

**FIGURE 2:**
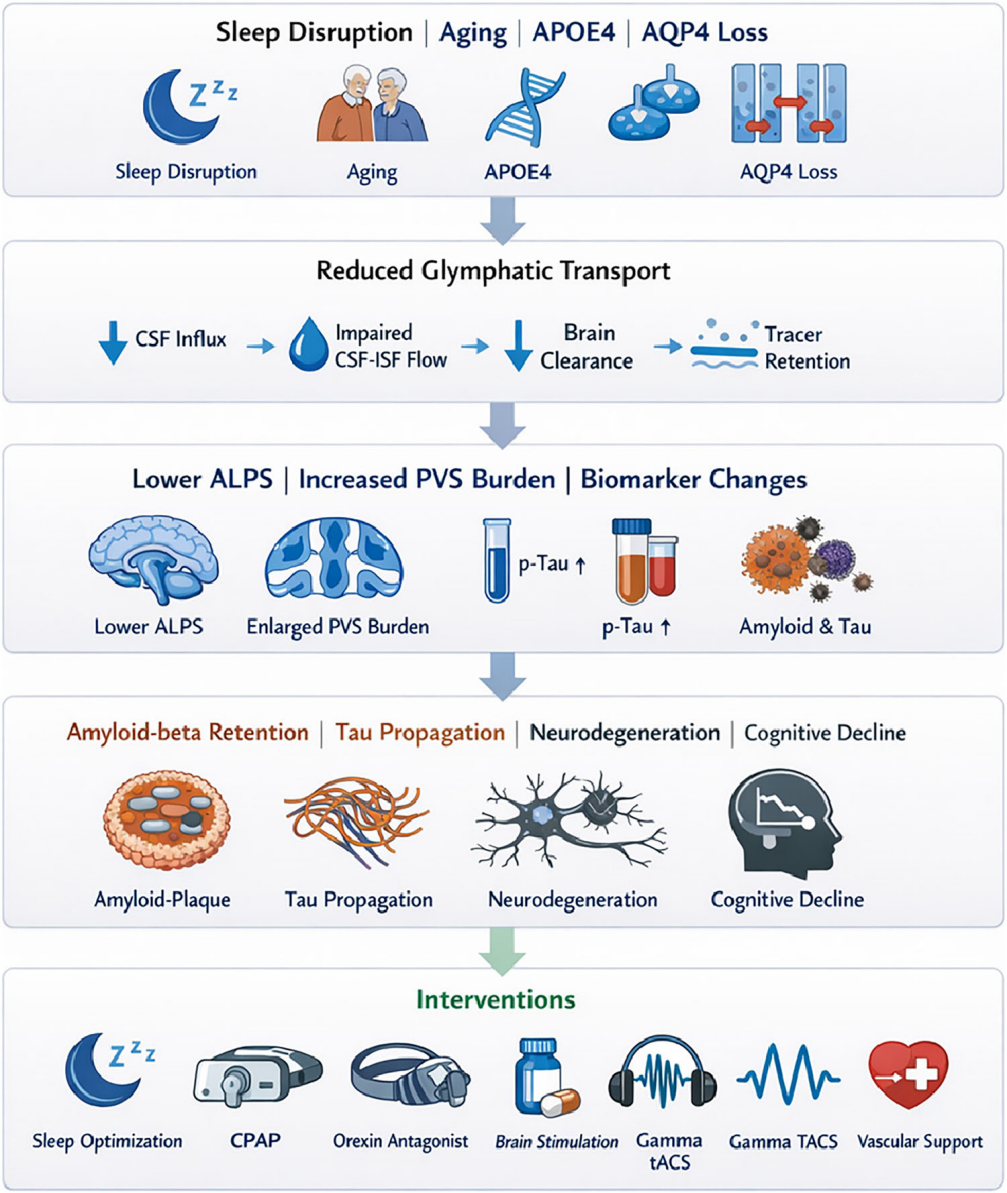
Sleep disruption, aging, vascular dysfunction, APOE4‐associated vulnerability, and AQP4 abnormalities may converge to impair glymphatic transport. This disruption is reflected in altered imaging and biomarker profiles, including lower ALPS values, greater perivascular space burden, abnormal tracer dynamics, and impaired clearance of amyloid‐beta and tau. These mechanisms also identify potential translational targets, including sleep‐based, pharmacologic, vascular, and neuromodulatory interventions.

A 2022 randomized pilot study of daily home‐based 40 Hz light and sound stimulation in mild probable AD reported safety, entrainment, and exploratory signals, including less ventricular expansion, less hippocampal atrophy, and improved associative memory relative to controls over three months (Chan et al. 2022). A 2024 sham‐controlled six‐month trial of an evoked gamma therapy system in mild to moderate AD likewise reported safety and high adherence, with nominal signals in some imaging and clinical outcomes but no definitive efficacy conclusions (Hajós et al. 2024). A 2025 open‐label extension in a very small cohort suggested two‐year feasibility and possible stabilization in some participants. In parallel, gamma transcranial alternating current stimulation has shown feasibility and preliminary cognitive benefits in prodromal or mild AD (Cantoni et al. 2025). These studies are exciting, but one should resist the temptation to over‐romanticize the 40 Hz wizardry. Most human trials do not directly measure glymphatic endpoints, so it remains unclear whether any observed benefit actually depends on enhanced clearance or whether immune, synaptic, or neurovascular mechanisms are doing most of the work (Fan and Gao 2025). A concise overview of completed and ongoing human intervention studies relevant to sleep, clearance biology, and AD is provided in Table 3 (Figure 2).

**TABLE 3:** Clinical trial and human intervention snapshot relevant to sleep, clearance biology, and Alzheimer's disease.

Intervention	Study design and population	Primary outcomes or biomarkers	Main finding	Status	Ref.
**Sleep versus sleep deprivation**	Randomized crossover, 39 adults	Plasma amyloid and tau species, sleep physiology modeling	Higher morning plasma amyloid and tau after sleep, interpreted as enhanced overnight clearance.	Completed and published	(Dagum et al. 2026)
**Suvorexant**	Randomized controlled trial, cognitively unimpaired adults	Serial CSF amyloid and tau phosphorylation	Acute dosing reduced CSF amyloid‐beta and a tau phosphorylation marker.	Completed and published	(Lucey et al. 2023)
**Closed‐loop acoustic stimulation**	At‐home intervention in AD patients	Slow‐wave activity and slow‐wave sleep	Enhanced slow‐wave activity at the group level, with marked interindividual variability.	Completed and published	(Van Den Bulcke et al. 2025)
**40 Hz audiovisual stimulation**	Randomized pilot in mild probable AD	Safety, entrainment, exploratory MRI, and cognition	Safe and feasible, with exploratory signals in memory and imaging.	Completed and published	(Chan et al. 2022)
**Evoked gamma therapy system**	Randomized sham‐controlled trial in mild to moderate AD	Safety, tolerability, exploratory clinical and MRI measures	Safe and well tolerated, but not powered for definitive efficacy.	Completed and published	(Hajós et al. 2024)
**Long‐term 40 Hz audiovisual stimulation**	Open‐label extension in mild AD	EEG entrainment, MRI, actigraphy, plasma pTau217	Two‐year feasibility with possible benefit signals in a very small sample.	Completed and published	(Chan et al. 2025)
**Gamma tACS**	Randomized sham‐controlled trial in prodromal or mild AD.	Cognition, EEG gamma power, plasma biomarkers	Feasible, with preliminary improvements during the controlled phase.	Completed and published	(Cantoni et al. 2025)
**Chronic 40 Hz light and sound therapy, NCT05655195**	Randomized sham‐controlled study in mild AD	EEG, blood biomarkers, MRI, cognition.	Designed to test sustained gamma entrainment effects on biomarkers and networks.	Active recruiting	(Chronic Treatment of Alzheimer's Disease With Gamma Frequency Stimulation 2022)

## 6 Clinical Implications

The sleep‐glymphatic relationship has several practical implications for AD prevention and management. First, sleep quality should be treated as part of risk assessment rather than as a peripheral lifestyle detail. Chronic insomnia, sleep apnea, circadian disruption, and fragmented sleep may represent modifiable contributors to long‐term proteostatic stress (Tahmasian et al. 2026). Second, clinicians should approach sleep treatment more carefully in older adults and at‐risk populations. The goal should not be sleep in the simplistic sense of sedation but the restoration of physiologically meaningful sleep architecture and continuity. This distinction becomes especially relevant in light of data suggesting that some hypnotics may suppress clearance‐related neurovascular dynamics (Schutte‐Rodin et al. 2008).

Third, biomarker development should become a major translational priority. A clinically useful glymphatic biomarker would ideally be noninvasive, reproducible, physiologically interpretable, and sensitive to intervention. At present, no single measure fully meets those criteria (Tomizawa et al. 2024). A multimodal strategy combining sleep EEG, fluid biomarkers, structural and functional MRI, and possibly emerging physiological tools may be more realistic. Fourth, clinical trials should incorporate clearance‐related endpoints directly (Einspänner et al. 2026). Trials of sleep interventions, orexin antagonists, gamma stimulation, and vascular or anti‐inflammatory therapies would be much more informative if they measured not only cognition and conventional biomarkers but also candidate readouts of clearance biology. Without that step, the field risks building a tower of inference on a somewhat wobbly basement (Herrero Babiloni et al. 2025).

## 7 Discussion

Several conclusions now seem reasonably robust. First, sleep alters the physical and neurovascular conditions that support CSF‐interstitial fluid exchange and solute transport. Second, sleep disruption affects amyloid and tau biology through a combination of impaired clearance and altered production or release. Third, direct impairment of glymphatic components, especially AQP4‐dependent pathways, can worsen proteopathic processes in animal models, including tau propagation (Lopes et al. 2024; Lucey et al. 2018). At the same time, important uncertainties remain. The first is measurement specificity in humans. DTI‐ALPS is useful and convenient, but it is biologically ambiguous. Some AD‐focused studies report strong associations with amyloid accumulation, neurodegeneration, and progression, whereas large population studies suggest the signal may primarily reflect vascular burden and white matter injury (Satpathi et al. 2025). Harmonized acquisition pipelines, validation against tracer methods, and better compartmental modeling will be necessary before ALPS can be treated as a reliable surrogate endpoint (Costea et al. 2025).

The second uncertainty concerns which aspects of sleep matter most. Many findings point to NREM slow‐wave physiology, but newer mechanistic work emphasizes dynamic norepinephrine signaling and vasomotion rather than sleep stage alone. This may explain why some sedatives fail to reproduce the clearance‐promoting features of natural sleep. Third, translation of oscillatory interventions remains incomplete (Bellesi et al. 2014). Gamma stimulation and acoustic slow‐wave enhancement are appealing because they are mechanistically informed and relatively noninvasive. Yet most human studies remain small, exploratory, and not powered to prove disease modification (Deng et al. 2024). More importantly, they rarely include direct clearance biomarkers. It is therefore premature to conclude that these interventions benefit AD by enhancing glymphatic transport, even if that hypothesis is plausible. Fourth, timing likely matters. Clearance biology appears to decline with aging, vascular stiffness, inflammation, and loss of AQP4 polarization (Deng et al. 2024). This suggests that preventive or very early interventions may be more effective than late‐stage treatment. It also implies that heterogeneity in vascular disease, sleep disorders, and baseline clearance capacity will complicate trial interpretation unless stratification is built into study design (Wei et al. 2025).

## 8 Conclusion

The sleep‐dependent glymphatic framework offers a compelling link between daily brain physiology and long‐term neurodegenerative risk. Experimental evidence strongly supports the view that sleep facilitates CSF‐interstitial fluid dynamics and promotes the removal of metabolites relevant to AD, while sleep loss disrupts this balance through effects on both clearance and protein homeostasis. More recent studies have sharpened this model by identifying norepinephrine‐driven vasomotion as a key driver of sleep‐associated clearance and by showing that human biomarker patterns may reflect overnight brain‐to‐blood transport during sleep.

For AD research, the most immediate implications are practical rather than magical. Preserving deep, continuous sleep, diagnosing and treating sleep disorders, and maintaining vascular health are reasonable near‐term priorities. For the field at large, the next major challenge is biomarker validation. Until human measures of glymphatic transport become sufficiently specific and reliable, therapeutic translation will remain suggestive rather than definitive. Even so, the concept has already changed how we think about the biology of sleep, proteostasis, and dementia. That alone is no small thing.

## Author Contributions

**Farshad Zare**: conceptualization, writing – original draft, writing – review and editing, supervision. **Gulnora Shakhmurova**: conceptualization, writing – original draft, writing – review and editing, supervision. **Jasur Rizaev**: writing – original draft, writing – review and editing. **Aseel Smeratz**: writing – original draft, writing – review and editing. **Rosull Saadoon Abbood**: writing – original draft, writing – review and editing. **Vinay Patil**: writing – original draft, writing – review and editing. **Manoj Kumar‐Mishra**: writing – original draft, writing – review and editing.

## Funding

The authors have nothing to report.

## Conflicts of Interest

The authors have no relevant financial or non‐financial interests to disclose.

## Data Availability

Data sharing is not applicable to this article as no datasets were generated or analyzed during the current study.
